# Gene expression responses of black spruce (*Picea mariana*) to global climate change conditions

**DOI:** 10.1186/1753-6561-5-S7-P99

**Published:** 2011-09-13

**Authors:** Om Rajora, Jinhong Kim, John Major, John Malcolm

**Affiliations:** 1 Faculty of Forestry and Environmental Management, University of New Brunswick, 28 Dineen Drive, Fredericton, NB E3B 5A3, Canada; 2 Natural Resources Canada, Atlantic Forestry Centre, 1350 Regent Street, Fredericton, NB E3B 5P7, Canada

## 

Global climate change conditions (elevated CO_2_ and atmospheric temperatures) are subjecting our forests, especially Boreal and temperate forests, to significant abiotic stresses, such as drought. This can affect health, productivity and fitness of our forests. Therefore, it is imperative to understand genomic and eco-physiological responses of forest trees to global climate change. We are addressing this aspect in black spruce (*Picea mariana*) - a transcontinental, ecologically and economically important tree species of the North American Boreal forest. Our objective was to determine gene expression and physiological responses and their inter-relationships in black spruce to elevated CO_2_, drought and co-stressed conditions.

We have used NGS whole transcriptome sequencing, cDNA-AFLP and qPCR analyses to identify, annotate and characterize genes expressed differentially in response to elevated CO_2_, drought and combined elevated CO_2_ and drought conditions in black spruce using the cloned material. Photosynthetic rate and stomatal conductance were measured simultaneously with tissue collection for RNA extraction. Thousands of transcripts (genes) showed differential expression (no expression, up-regulation or down-regulation) in response to elevated CO_2_, drought and/or their combined conditions, with over 1600 genes from several pathways showing >10-folds gene expression differences between control and treated plants. A number of genes showed 100 to 500 folds up or down regulation in response to elevated CO_2_, drought or their combined conditions. Responses to each treatment at the gene expression and physiological levels were correlated well among different genotypes. We will present these results which contribute significantly to our understanding of tree’s responses to global climate change.

